# Fibroblast α11β1 Integrin Regulates Tensional Homeostasis in Fibroblast/A549 Carcinoma Heterospheroids

**DOI:** 10.1371/journal.pone.0103173

**Published:** 2014-07-30

**Authors:** Ning Lu, Tine V. Karlsen, Rolf K. Reed, Marion Kusche-Gullberg, Donald Gullberg

**Affiliations:** 1 Department of Biomedicine, University of Bergen, Bergen, Norway; 2 Centre for Cancer Biomarkers, Norwegian Centre of Excellence, University of Bergen, Bergen, Norway; University of Patras, Greece

## Abstract

We have previously shown that fibroblast expression of α11β1 integrin stimulates A549 carcinoma cell growth in a xenograft tumor model. To understand the molecular mechanisms whereby a collagen receptor on fibroblast can regulate tumor growth we have used a 3D heterospheroid system composed of A549 tumor cells and fibroblasts without (α11^+/+^) or with a deletion (α11^-/-^) in integrin α11 gene. Our data show that α11^-/-^/A549 spheroids are larger than α11^+/+^/A549 spheroids, and that A549 cell number, cell migration and cell invasion in a collagen I gel are decreased in α11^-/-^/A549 spheroids. Gene expression profiling of differentially expressed genes in fibroblast/A549 spheroids identified CXCL5 as one molecule down-regulated in A549 cells in the absence of α11 on the fibroblasts. Blocking CXCL5 function with the CXCR2 inhibitor SB225002 reduced cell proliferation and cell migration of A549 cells within spheroids, demonstrating that the fibroblast integrin α11β1 in a 3D heterospheroid context affects carcinoma cell growth and invasion by stimulating autocrine secretion of CXCL5. We furthermore suggest that fibroblast α11β1 in fibroblast/A549 spheroids regulates interstitial fluid pressure by compacting the collagen matrix, in turn implying a role for stromal collagen receptors in regulating tensional hemostasis in tumors. In summary, blocking stromal α11β1 integrin function might thus be a stroma-targeted therapeutic strategy to increase the efficacy of chemotherapy.

## Introduction

The tumor stroma is composed of a network of extracellular matrix molecules and associated cells, which interact in a reciprocal manner. It is now well recognized that the tumor stroma plays an important part in the growth of most solid tumors by directly and indirectly affecting different aspects of tumorigenesis and tumor angiogenesis [1], [2], [3].

Collagen is a major extracellular matrix (ECM) molecule in the stroma of many carcinomas. Recent work has convincingly shown that the stiffness of the tumor stroma, and in particular that mediated via collagen cross-linking, in a β1 integrin- and Erk-dependent manner can regulate tumor cell growth [4]. In addition to the organization of the interstitial collagen affecting physical properties of the tumor microenvironment, the actomyosin–dependent contractility of cells within the tumor stroma tissue affects the physical properties of the stromal compartment to regulate tensional homeostatis [5], [6].

When present in the stroma of solid tumors fibroblasts are often activated and are called cancer-associated fibroblasts (CAFs). Since it is now widely accepted that fibroblasts are heterogeneous, the nature of the molecules involved in different tumor cell-stroma interactions vary in a tissue-specific manner [3]. In normal connective tissues, fibroblasts contribute to the interstitial fluid pressure in an integrin-dependent manner [7], [8], [9]. In the tumor context, a collagen-rich tumor stroma populated by contractile CAFs contributes to the interstitial fluid pressure in the tumor and serves as a severe barrier to chemotherapy approaches [10], [11].

A variety of approaches have been used to study tumor-stroma interactions *in vitro* and *in vivo* in trying to understand the complex nature of the molecular interplay in tumors. Heterotypic stromal tumor spheroids offer an opportunity to study tumor-stroma interactions under 3D conditions with clear advantages over 2D co-cultures in recapitulating a more *in vivo* like microenvironment in terms of cell-cell and cell-matrix interactions [12], [13], [14]. It has been shown that fibroblasts in a 3D system produce other factors then when grown in 2D environment [15], [16] and that the spheroid 3D milieu is well suited for chemoresistance studies of cancer- and cancer stem cells [17], [18], [19].

Integrin α11 is a collagen-binding mesenchymal integrin subunit, which we previously have shown to be up-regulated in the non-small cell lung cancer (NSCLC) stroma [20]. Functional analyses have identified α11β1 as a major collagen receptor on mouse embryonic fibroblasts (MEFs) [21], [22]. α11 is up-regulated by TGF-β and the increased expression level is in part dependent on the presence of a Smad-binding element in the α11 promoter [23]. In addition, the expression level of α11 integrin in MEFs is sensitive to the mechanical stiffness of the environment in a mechanosensing mechanism, which involves an autocrine loop of Activin A [24]. In cardiac fibroblasts plated on glycated collagen I, the autocrine loop regulating α11 levels involves TGF-β2 [25]. By a so far uncharacterized mechanism, α11β1 integrin appears to regulate myofibroblast differentiation on collagen substrates [24], [25].

Recent analysis of NSCLC cell lines have identified TGF-β dependent mechanisms of bi-directional communication between carcinoma cells and fibroblasts, involving a central role for αvβ6 on carcinoma cells in activating TGF-β and initiating activation of fibroblasts [26]. In the recent study A549 cells were found to express low levels of αvβ6 [26] and they thus offer an opportunity to study tumor-stroma interactions without the involvement of carcinoma-derived active TGF-βWhereas data has accumulated on the type of molecules involved in inter-cellular communication in the tumor microenvironment, less is known about the molecular mechanisms involved in the control of interstitial fluid pressure in tumors. The potential role/s of fibroblast collagen receptors in controlling tensional homeostasis in the tumor microenvironment is as of yet poorly understood.

We have recently shown that the interstitial fluid pressure is reduced in heterospheroids composed of tumor cells and mouse fibroblasts deficient in heparan sulfate proteoglycan synthesis [27]. In the current study, we have taken advantage of genetically engineered fibroblasts lacking α11 integrin [21] in a similar mixed spheroid system with A549 carcinoma cells to dissect the detailed mechanisms whereby α11β1 on fibroblasts affect tumor cell proliferation and cell migration. Using microarray we identify CXCL5 as a factor regulated in A549 cells interacting with α11β1-expressing fibroblasts. Furthermore, we suggest an important role for α11β1-dependent collagen remodeling in regulating the interstitial fluid pressure within the spheroids.

## Materials and Methods

### Cell Culture

SV40-immortalized MEFs were derived from wild-type (α11^+/+^) and Itga11 knock-out (α11^-/-^) mouse embryos at E14.5 as described previously [21]. In order to restore the function of integrin α11, full-length human ITGA11 cDNA was transfected into SV40-immortalized α11^-/-^ MEFs (α11^-/-/α11^) as described previously [28]. The human non-small cell lung adenocarcinoma A549 cell line was purchased from the American Type Culture Collection (ATCC). Monolayer cells and multicellular spheroids were cultured in Dulbeccos modified Eagles medium (DMEM) with Glutamax (Gibco) supplemented with 10% fetal bovine serum (FBS), 100 units/ml of penicillin and 0.1 mg/ml of streptomycin (all from PAA Laboratories).

### Spheroid preparation and culture

Single cell type multicellular spheroids (homospheroids) and composite spheroids containing a mixture of MEFs and tumor cells (heterospheroids) were prepared using the hanging drop method as described previously [27]. Briefly, sub-confluent cells were trypsinized and suspended in culture medium to a concentration of 1×10^6^/ml. The MEF and A549 cell suspensions were then mixed at a ratio of 4∶1. Approximately 40 drops (25 µl/drop, 2.5×10^4^ cells) were dispensed onto a lid of a cell culture dish. The lid was then inverted and placed over a cell culture dish containing DMEM for humidity, and cultured under standard conditions. The liquid overlay technique was used in the beginning of the study to compare with the hanging drop method. Briefly, confluent monolayers were trypsinized, resuspended in the cell culture medium to a concentration of 3×10^6^ cells/ml. Single cell type suspension or the mixture of MEF and A549 (4∶1) cell suspension was re-plated in a drop-wise fashion to 10-cm cell culture dishes pre-coated with 0.75% agar (Noble agar, Difco). The spheroids were usually grown for 6 days and the culture medium were renewed on day 3.

### Western-blot analysis

Western blotting was performed as described previously [23]. The primary rabbit anti-mouse and rabbit anti-human α11 antibodies [29] were used at a dilution of 1∶500, and the mouse anti-β-actin antibody at a dilution of 1∶5000. The secondary goat anti-rabbit and goat anti-mouse HRP-conjugated antibodies (Santa Cruz Biotechnology) were applied at a dilution of 1∶5000. Chemiluminescence signals were developed using the ECL Western-blotting systems kit (GE Healthcare) and photographed using the ChemiDoc XRS device and the Quantity One 1-D Analysis Software (Bio-Rad).

### Immunofluorescence (IF) staining of spheroids

IF staining was performed on cryosections of 4 and 6 days old heterospheroids as described previously [27]. Primary Rabbit anti-mouse collagen type I monoclonal antibody (1∶200; AB765P, Millipore), rat anti-mouse β1 integrin monoclonal antibody (1∶400; MAB1997, Millipore) and rabbit anti-human cytokeratin-7 polyclonal antibody (1∶400; NBP1-30152, Novus Biologicals) were used to stain MEFs and A549 cells, respectively. Secondary antibody DyLight 488- AffiniPure Goat Anti-Rat IgG (1∶800; 112-485-143, Jackson ImmunoResearch) and DyLight 549-AffiniPure Goat Anti-Rabbit IgG (1∶800; 112-505-167, Jackson ImmunoResearch) were used for β1 integrin and cytokeratin-7, respectively. Sections stained with secondary antibody only were used as negative controls. The stained sections were observed under a Zeiss Axioscope fluorescence microscope and photographed using a digital AxioCam mRM camera (Zeiss).

### Collagen gel contraction assay

Collagen I gel solution (10 ml, final concentration 1.2 mg/ml) was prepared by mixing 5 ml 2×DMEM, 1 ml 10×HEPES (0.2 M, pH 8.0) and 4 ml collagen type I (3 mg/ml, PureCol, Advanced Biomatrix). All solutions were kept on ice before and during mixing. Cell suspensions were prepared to a concentration of 1×10^6^ cells/ml and added to the collagen solution to get a final density of 1×10^5^ cells/ml. The collagen-cell mix aliquots (100 µl/well) were then added to a 96-well plate and gels were allowed to polymerize in the cell culture incubator for 1 h 30 min. Gels were floated by adding 100 µl/well of DMEM with 4% FCS after polymerization. Free-floating gels continued to be incubated at 37°C and collagen gel contraction were monitored by measuring the gel diameter under an inverted microscope at different time points.

### Interstitial fluid pressure measurements

The interstitial fluid pressure (P_if_) measurements of the spheroids were performed as described previously [27]. Briefly, the spheroids were collected and transferred to 10-cm Lysine-coated cell culture dishes (NUNC) and left to attach for 2 h at 37°C. P_if_ was measured by micropuncture technique using sharpened glass capillaries (tip diameter 3–5 µm) filled with 0.5 M NaCl colored with Evans blue dye connected to a servo-controlled counter pressure system. The glass capillary was inserted into the spheroid with the help of a stereomicroscope (Wild M5, Heerbrugg, Switzerland). P_if_ measured in the cell culture medium immediately outside the spheroid was defined as its zero pressure. P_if_ inside the spheroid was recorded for 30 seconds [30].

### Spheroid migration assay on collagen

Glass coverslips in a 24-well plate were coated overnight at 4°C with 100 µg/ml of bovine collagen type I (PureCol, Advanced Biomatrix). Spheroids of 6 days old were seeded onto the coverslips and incubated at 37°C. Cell migration out from the spheroids was observed under an inverted phase contrast microscope (Leica DMIL) and photographed after 4 h and 24 h. After 24 h the coverslips were fixed with methanol for 5 min at −20°C and subjected to immunofluorescence staining as described above in the section of **Immunofluorescence staining (IF) of spheroids**.

### Spheroid migration in 3D collagen gel

Collagen gels were prepared one day prior to the seeding of spheroids as described in the collagen gel contraction assay. Instead of mixing cells with the collagen solution, 1–3 spheroids were embedded into the collagen gel by pipetting them into the gel solution after the gel solution were added 100 µl/well to a 96-well plate. The collagen-spheroid mixtures were then left to polymerize in the cell culture incubator. In order to establish the possible contribution of TGF-β1, the TGF-β1 inhibitor SB431542 (Sigma) was added to the mixtures with the final concentration of 1 µM. Cell migration from spheroids embedded in collagen gels was monitored under an inverted light microscope (Leica DMIL) and photographed at different time points.

### A549 proliferation in the heterospheroids

A549 proliferation in the heterospheroids was tested using a luciferase assay as described previously [27]. Briefly, A549 cells stably expressing firefly luciferase (A549-Luc) were used to prepare different types of heterospheroids as described above. Spheroids from 20 hanging drops (prepared with 2.5×10^4^ cells/drop) were collected from day 2 to day 6. Five hundred microliters of cells mixture (5×10^5^ cells) were also collected when preparing the heterospheroids as the starting point for the assay (day 0). Collected cell mixtures and spheroids were frozen at -70°C until samples for all time points were collected and applied to luciferase analysis.

### Spheroid RNA extraction and microarray analysis

All the spheroid samples were collected and stored at −80°C before they were subjected to total RNA isolation. The total RNAs were isolated from the samples using RNeasy Mini Kit (Qiagen) according to the manufacturer's instructions.

Extracted total RNA was quality tested on Agilent Bioanalyzer 2100 and microarray was performed at the NMC-UoB Microarray Core Facility using the Illumina Bead Array Technology (HumanHT-12 v4 Expression Bead Chip). The raw microarray data was quality examined in GenomeStudio and SampleProbeProfile-text file was exported from GenomeStudio, during which, control probes were removed. The raw data is available at ArrayExpress (http://www.ebi.ac.uk/arrayexpress/) with accession number E-MTAB-2687. The resulting gene expression table was imported into J-Express 2012 (http://jexpress.bioinfo.no/site/) for further quality control and analysis. Differentially expression genes between the two groups of samples (RNAs from α11^+/+^/A549 vs α11^-/-^/A549) were analyzed using SAM method and only the genes with q-value less than 10% were considered to be valid.

### Real-time quantitative Reverse Transcription PCR (qRT-PCR)

The qRT-PCR was performed as described previously (Carracedo, 2010). cDNA was generated from 1 µg of total RNA, prepared as described above, using M-MuLV reverse transcriptase (Fermentas) and oligo (dT)_18_ primer. For each sample, 100 ng of transcribed cDNA and 0.5 µM of each primer were used as template in 20 µl PCR reaction using iQ SYBR Green Supermix (170–8893; Bio-Rad). qRT-PCRs were performed in a LightCycler 480 Instrument II (Roche Applied Science). The fold change of the gene expression was calculated using the 2^-ΔΔCt^ method. PCRs were performed in triplex for each cDNA sample and negative controls with no template were included for each primer pair. The primer sequences are listed in **Table S1**.

### Statistical Analysis

Statistical analyses were performed to evaluate differences between groups (n). For comparisons where n>2, a two-way Anova with Bonferroni-Dunn correction for multiple comparisons, was applied. For comparisons where n = 2, the two-tailed, unpaired t-test was performed. For both methods, P<0.05 was considered statistically significant.

## Results

### Integrin α11β1 affects heterospheroid size

**Figure 1:**
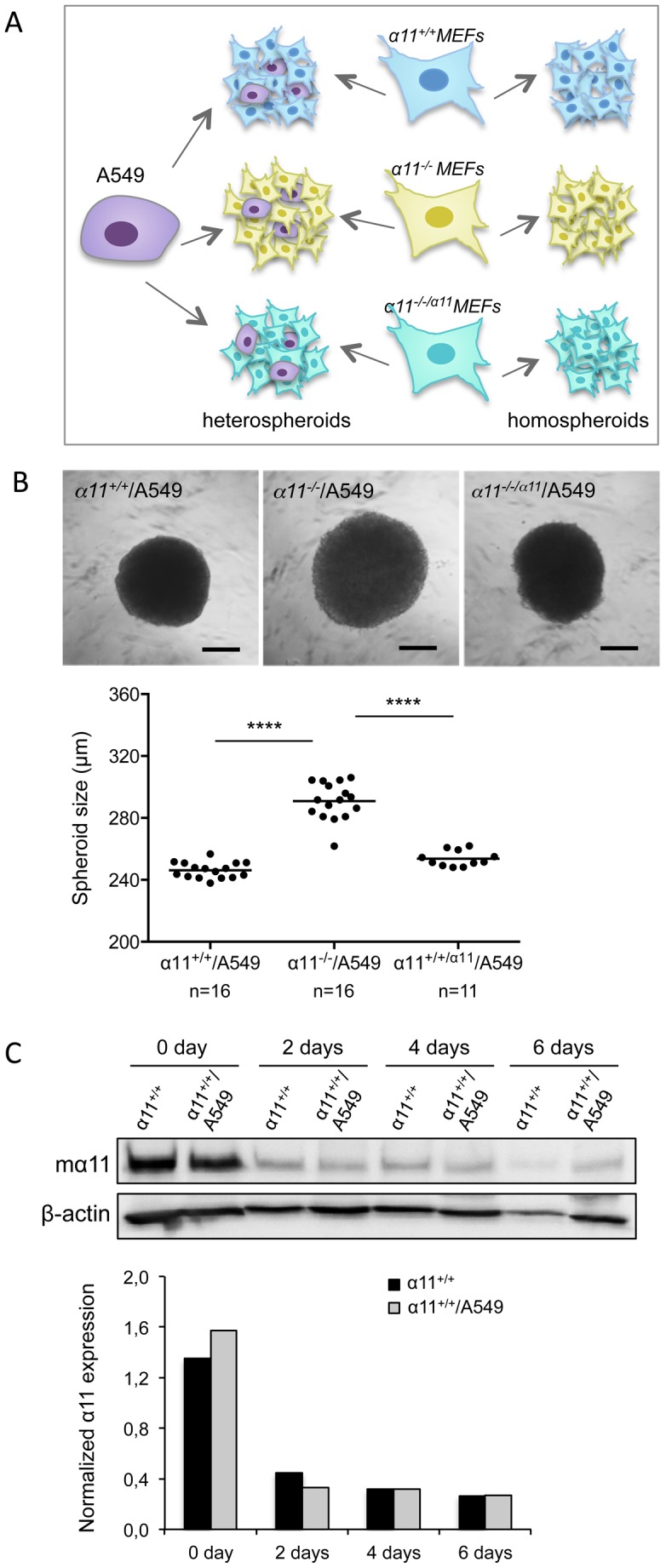
Integrin α11 expression on MEFs affects heterospheroid size. (A) Schematic illustration of spheroid formation: Spheroids were grown from fibroblast cells alone (homospheroids), or mixed with A549 tumor cells in a ratio of 4∶1 (MEFs: A549) to form heterospheroids. (B) Representative phase contrast images of heterospheroids generated by the hanging drop method after 4 days in culture. Heterospheroids composed of α11^-/-^ MEFs formed larger spheroids. The size difference is significant between the α11^-/-^/A549 and the α11-containing spheroids (α11^+/+^/A549 and α11^-/-/α11^/A549). Size bars  =  100 µm; ****p< 0.0001. (C) Western-blot analysis of α11 integrin expression level during spheroid culture at the indicated time points. The lower panel shows the α11 integrin expression levels after normalizing to β-actin expression levels.

We initially used the liquid overlay method to generate spheroids composed of either MEFs alone (homospheroids) or mixture of MEFs and A549 cells (heterospheroids; MEFs/A549). However, the spheroids formed by this method showed large variation in size and shape between preparations and within one plate from the same preparation. Therefore, we instead used the hanging drop method which resulted in spheroids which were homogenous in size and shape [27]. **Fig. 1A** illustrates the different types of MEF homospheroids and MEF/A549 heterospheroids used in the present study, where the MEFs and A549 cells were mixed in a ratio of 4∶1. There was no apparent size difference between the MEF homospheroids of different genotypes (**data not shown**), while there was a significant size difference between the knockout α11^-/-^/A549 spheroids and the wildtype α11^+/+^/A549 as well as between α11^-/-^/A549 and knock-in α11^-/-/α11^/A549 spheroids (**Fig. 1B**). The α11^-/-^/A549 spheroids were larger and seemed to be less compact than the other two types of heterospheroids. The expression of integrin α11 in the α11^+/+^ MEFs and the α11^-/-/α11^ MEFs was verified by Western blotting (**Fig. S1**). α11 expression was constant within the α11^+/+^/A549 spheroids from day 2 for the time period studied (**Fig. 1C**). Since α11 expression is mechanosensitive we believe that the initial high expression of α11 in 3D spheroids is due to previous culturing on the stiff plastic surface.

### Fibroblasts in heterospheroids synthesize collagen I

**Figure 2:**
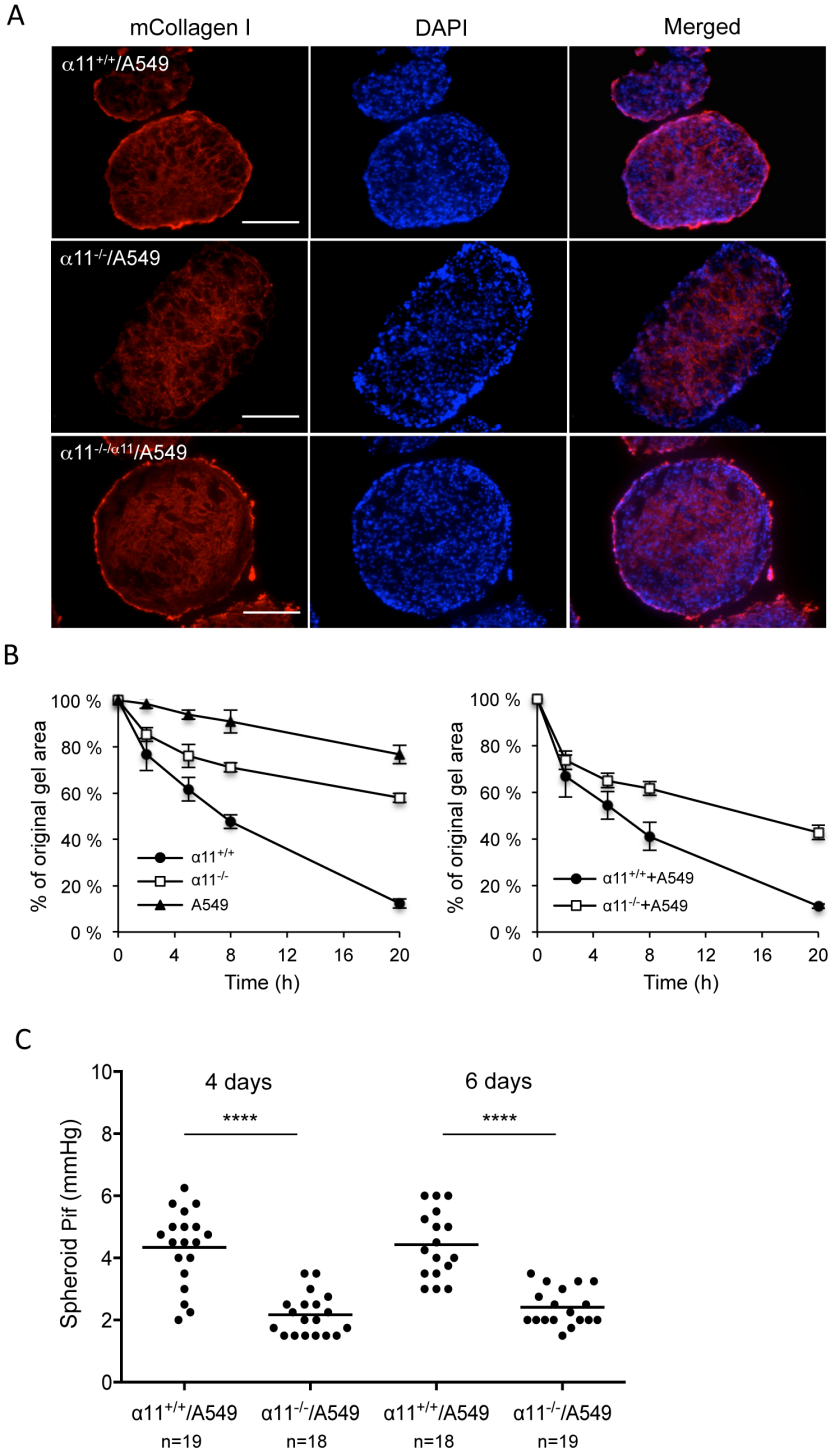
Absence of α11 expression on MEFs leads to impaired ability to contract collagen I lattices and decreased interstitial fluid pressure in heterospheroids. (A) Heterospheroids of MEFs of different genotypes and A549 (as indicated) were fixed and stained with an antibody towards mouse collagen type I (red). Nuclei were counterstained with DAPI (blue). Size bars  =  100 µm. (B) A549 cells or the MEFs and A549 cell mixtures (MEFs+A549, ratio 4∶1) were embedded in type I collagen as indicated. Contraction of collagen gels was monitored at 2, 4, 8 and 20 hours. Three independent experiments were performed and data shown are from one representative experiment with 6-8 replicates for each cell type or cell mixture. Error bars represent standard deviation. (C) Interstitial fluid pressure measurement of the spheroids. The interstitial fluid pressure (P_if_) was measured in spheroids cultured for 4 and 6 days. The numbers of spheroids measured (n) are as indicated. **** p< 0.0001.

Since the α11^-/-^/A549 spheroids appeared less compact, we asked if the absence of integrin α11 could affect the ability of MEFs to synthesize or organize a collagen matrix. Immunostaining showed that collagen I was expressed by MEFs of both genotypes (**Fig. 2A**). The capacity of cells to reorganize the matrix was monitored by a collagen gel contraction assay where MEFs alone or cell mixtures of MEFs and A549 cells (MEFs+A549, ratio 4∶1) were seeded into a collagen I matrix. The gel contraction assay demonstrated that the α11^-/-^ MEFs alone and α11^-/-^ MEFs mixed with A549 cells (α11^-/-^ +A549) displayed reduced capacity to contract a collagen gel in comparison with α11^+/+^ MEFs alone and α11^+/+^ MEFs mixed with A549 cells (α11^+/+^ +A549) (**Fig. 2B**), indicating that the collagen matrix in spheroids may also be differently reorganized depending on the presence or absence of fibroblast α11β1 integrin.

### α11β1-dependent regulation of interstitial fluid pressure

Since α11^-/-^/A549 spheroids appeared less compact than the α11^+/+^/A549 and α11^-/-/α11^/A549 spheroids, together with the finding that α11β1 is essential for collagen reorganization, we measured the interstitial fluid pressure (P_if_) in the different spheroids. P_if_ measurements were performed on 4-day- and 6-day-old α11^+/+^/A549 and α11^-/-^/A549 spheroids. The results demonstrated that P_if_ in α11^-/-^/A549 spheroids was significantly lower than in α11^+/+^/A549 spheroids at both time points (**Fig. 2C**), suggesting that α11β1-mediated contraction of collagen matrix contributes to the increased interstitial fluid pressure in spheroids.

### α11-dependent A549 cell migration and proliferation within spheroids

**Figure 3:**
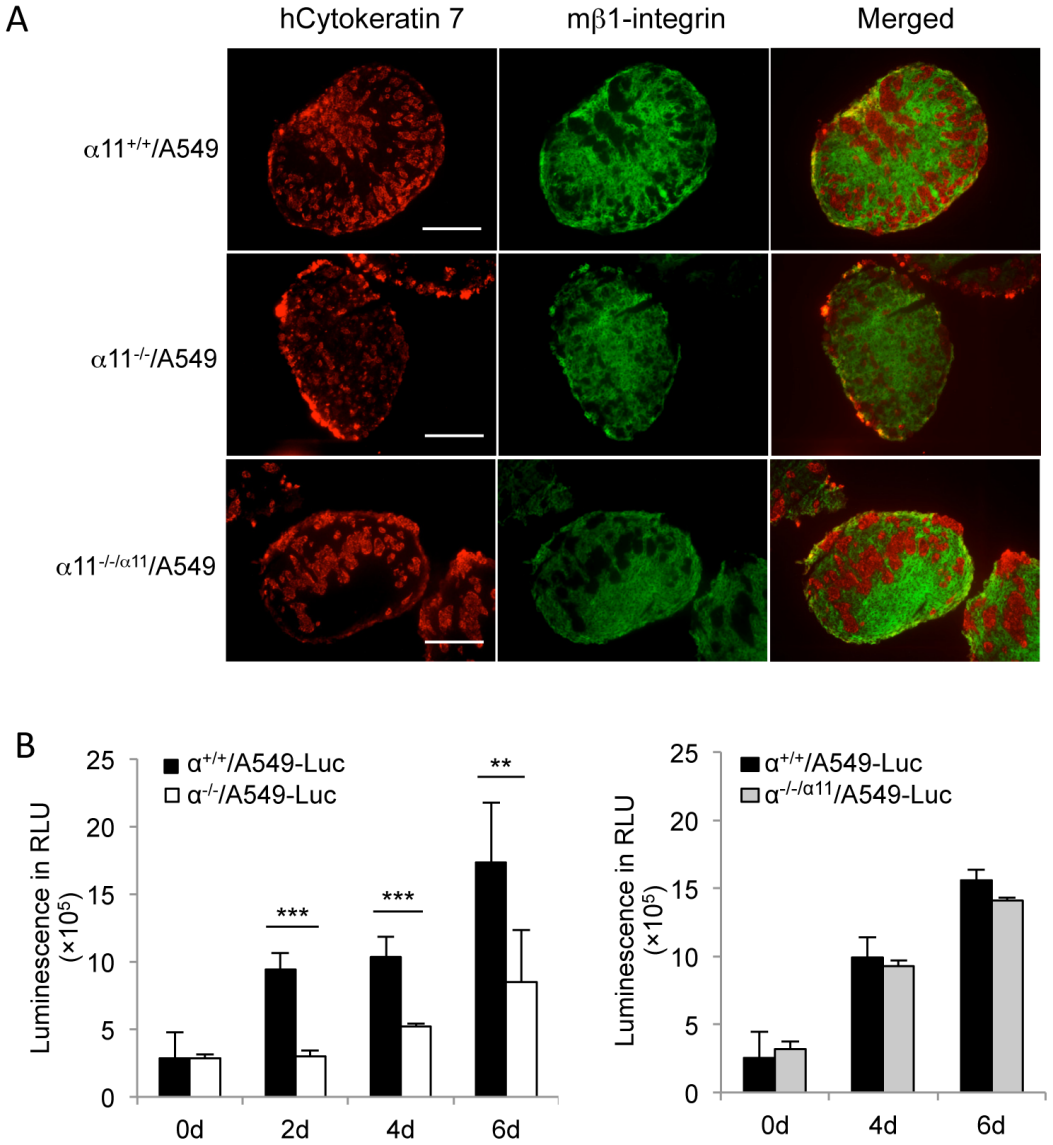
A549 cell segregation and proliferation inside the spheroids. (A) Four- day-old heterospheroids (as indicated) were double-stained with anti-human cytokeratin 7 (stained A549 cells, red) and anti-mouse β1 integrin (stained MEFs, green) antibodies. Size bars  =  100 µm. (B) A549 proliferation in the spheroids. Spheroids were prepared with different types of MEFs and A549 cells stably expressing the firefly luciferase (A549-Luc). A549 proliferation was measured by luciferase assay on spheroids at different time points. Each experiment was performed with spheroid collected from three separate preparations at each time point and measured in duplicte. Experiments were repeated for at least three times and shown is the representative result from one experiment. Error bars indicate standard deviation. ** p<0.01, *** p<0.001.

Immunofluorescence staining of the 4-day-old spheroids showed that the A549 cells and MEFs tended to segregate with time in the spheroids so that A549 migrated towards the periphery of the spheroids. There was a clear trend for a slower segregation of cells in the α11^-/-^/A549 spheroids (**Fig. 3A**). Occasionally, all the A549 cells in the α11^+/+^/A549 spheroids had migrated to the outer layer of the spheroids at 6d, whereas the segregation was incomplete in parallel spheroids containing α11^-/-^ MEFs (**Fig**. **S2**). The staining patterns in **Fig. 3A** indicated less tumor cells in the α11^-/-^/A 549 spheroids after 4 days compared to the α11^+/+^/A549 spheroids. To determine if this was due to a reduced proliferation rate, we determined the effect of MEFs on A549 cell proliferation inside heterospheroids, using A549 cells transduced with lentiviral vector expressing Luciferase (A549-Luc). The results convincingly demonstrate that A549-Luc cells proliferate at a lower rate in the α11^-/-^/A549 spheroids compared to the α11^+/+^/A549 spheroids, while there was no significant difference between A549 proliferation in the α11^+/+^/A549 and in the α11^-/-/α11^/A549 spheroids (**Fig. 3B**).

### α11-dependent A549 cells migration onto a collagen type I monolayer

**Figure 4:**
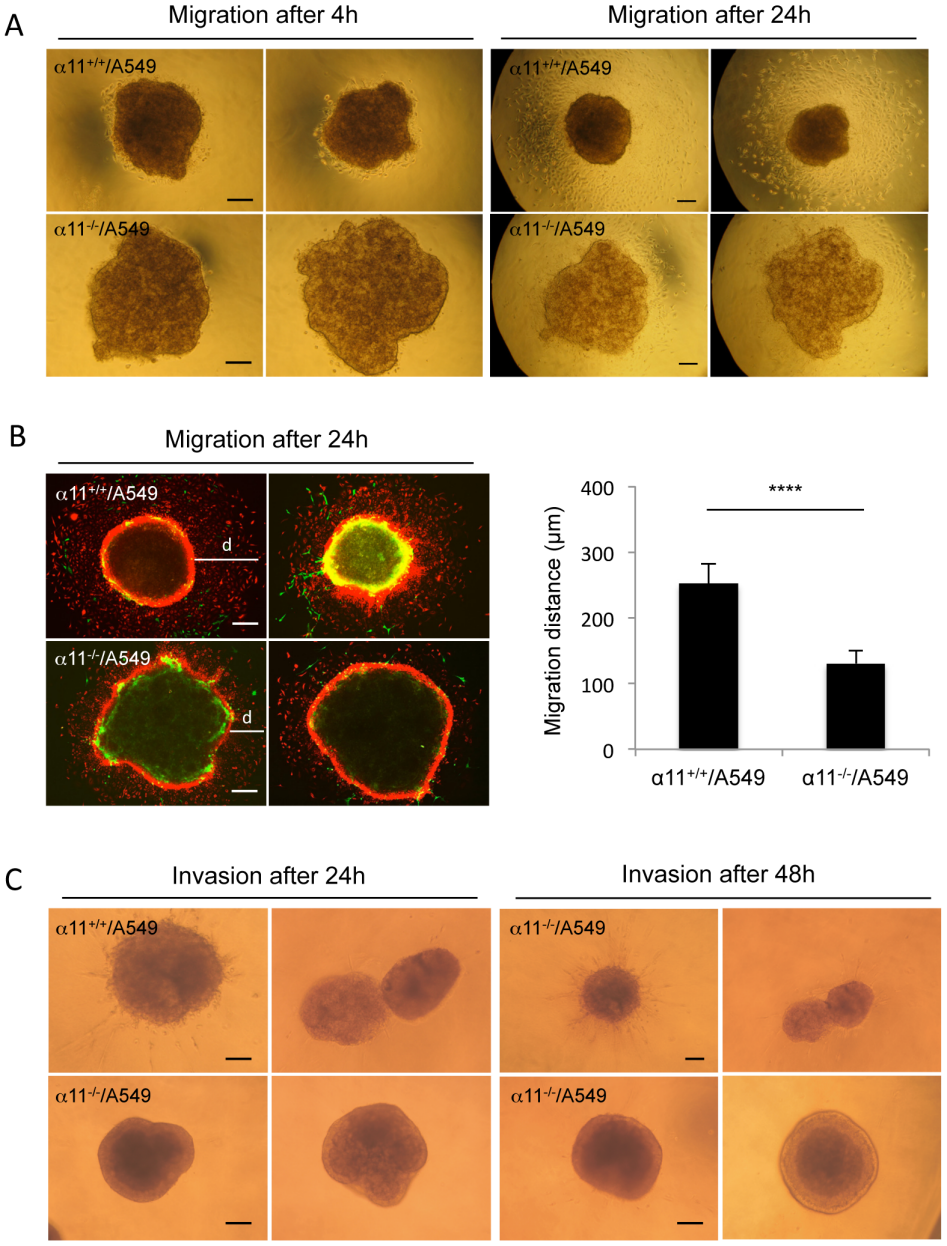
Spheroid migration on collagen I monolayers and invasion in 3D collagen gels. (A) A549 migration out from the heterospheroids. Six-day-old heterospheroids were seeded on collagen type I-coated coverslips and cultured under conditions as described in Materials and Methods. Migration of the cells from the spheroids was examined under an inverted phase contrast microscope 4 and 24 hours after the spheroids were seeded. Size bars  = 100 µm. (B) Fluorescence immunostaining of the spheroids on coverslips after 24 h and migration distance of A549 from the heterospheroids. Spheroids were double stained with antibodies towards human keratin 7 (stained A549 cells, red) and mouse β1 integrin (stained MEFs, green). Six of each type of spheroids were stained. The maximum migration distance (d) of A549 cells were measured on the photographs as indicated in the figure and calculated according to size bar. Size bars  = 100 µm. **** p<0.0001. (C) Spheroid invasion in 3D collagen I gel. Six to eight 6-day-old spheroids were embedded in 3D collagen gels (1 mg/ml collagen type I) and invasion of cells was observed and photographed under an inverted phase contrast microscope after 24 h and 48 h. Two representative images are shown. Size bars  = 100 µm.

The migratory ability of A549 cells in six-day-old α11^+/+^/A549 and α11^-/-^/A549 spheroids were further tested by a spheroid migration assay on collagen I. Four hours after seeding spheroids onto collagen gels, a cell migration ring from α11^+/+^/A549 spheroids appeared, while only few cells started to migrate out from α11^-/-^/A549 spheroids, at this time point (**Fig. 4A**). After 24 h, cells from α11^-/-^/A549 spheroids migrated a significantly shorter distance than those from α11^+/+^/A549 spheroids (**Fig. 4A**). Immunostaining showed that the cells that had migrated out from the spheroids were mainly A549 cells (**Fig. 4B**), while only few MEFs had left the spheroids. These data suggest that MEF/A549 interactions regulate the migratory capacity of the tumor cells.

### α11-dependent A549 invasiveness inside a 3D collagen gel

To further examine the influence of fibroblast α11 on the migratory and invasive capacity of A549 cells, α11^+/+^/A549 and α11^-/-^/A549 spheroids were imbedded into a 3D collagen type I gel and cells were allowed to migrate and invade the collagen gel for up to 48 hours. Very strikingly, after 24 h and 48 h only cells from α11^+/+^/A549 spheroids invaded the collagen gel (**Fig. 4C**). To test the possible involvement of TGF-β signaling, the ALK 4,5,7 inhibitor SB431542 (1 µM) was included in the assay. However, SB431542 had no effect on the cell invasion ability from α11^+/+^/A549 spheroids under the conditions shown, which is in agreement with recent data demonstrating lack of endogenous TGF-β signaling axis in A549 cells [26] (**Fig. S3**). These data suggest that the invasive capacity of the A549 cells is influenced byα11β1-dependent paracrine events.

### Gene expression profiling of A549 cells in the α11^+/+^/A549 and α11^-/-^/A549 heterospheroids

**Figure 5:**
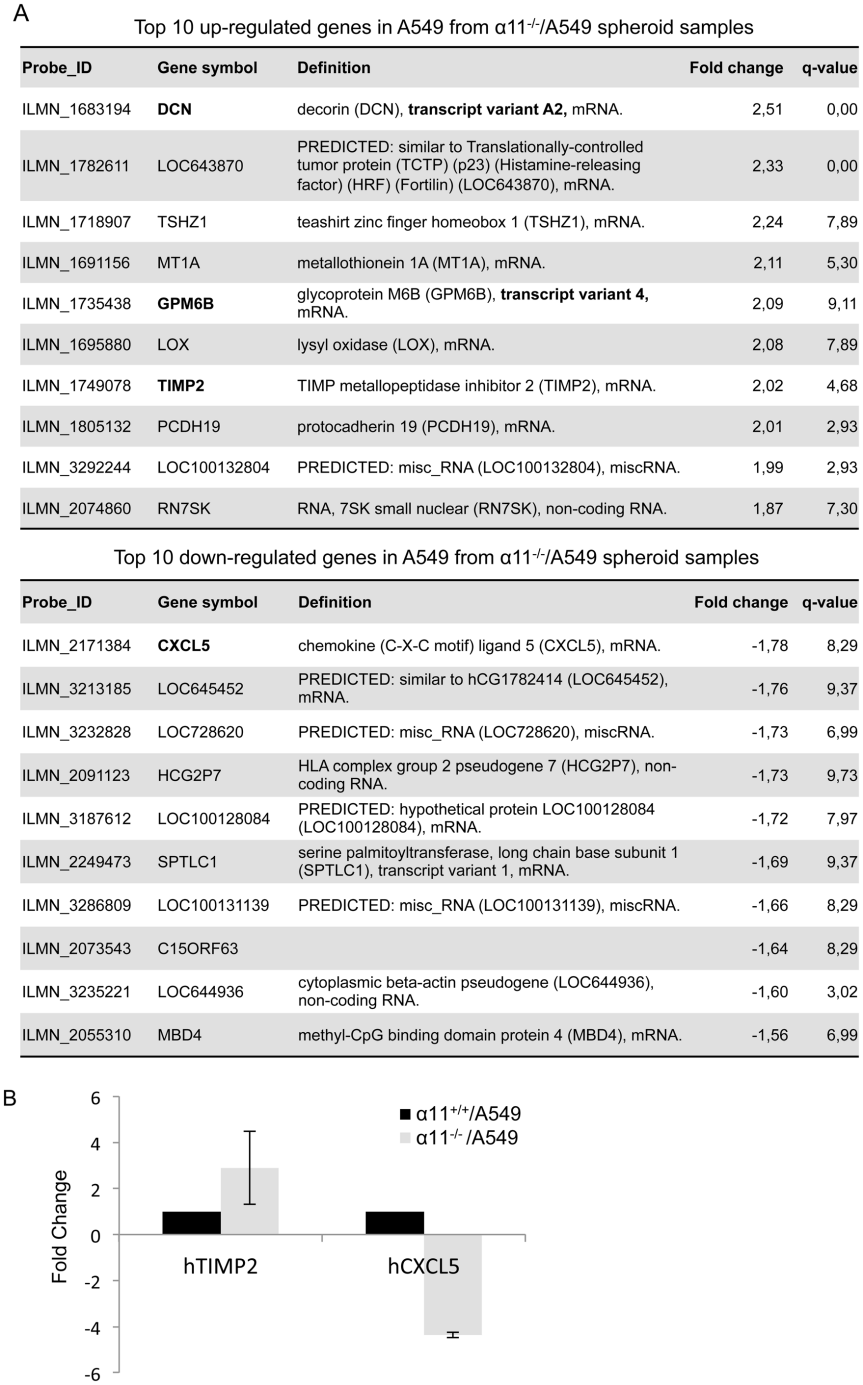
A549 expression profiling in α11^-/-^/A549 and α11^+/+^/A549 heterospheroids. (A) The top 10 up- and down-regulated genes in A549 cells in α11^-/-^/A549 spheroids compared with those in α11^+/+^/A549 spheroids. Fold changes shown are the average values from 6 individual samples from each type of spheroid. Gene symbols marked in bold are the genes chosen for RT-qPCR verification. (B) Microarray data verification of two selected genes using qRT-PCR. Total RNA was extracted from 6-day-old spheroids prepared in new replicate experiments in conditions identical to those used for Microarray (n = 6). Shown is the fold change of the mRNA expression levels of the genes in α11^-/-^/A549 comparing with those in α11^+/+^/A549 spheroids (set arbitrarily as 1). Error bars represent standard deviation across 6 independent samples.

To explore the mechanism(s) underlying the observed differences in spheroid morphology and tumor cell behavior between the α11^-/-^/A549 and α11^+/+^/A549 spheroids, microarray analysis was performed to compare the gene expression profiles of A549 cells in α11^-/-^/A549 and α11^+/+^/A549 heterospheroids. J-Express analysis of the microarray data revealed a number of genes that were differentially expressed in A549 cells from α11^-/-^/A549 spheroids as compared with α11^+/+^/A549 spheroids. Among the 160 differentially expressed genes (selected by SAM method, q<10%), most of the genes (136 genes) were up-regulated and much fewer genes (24 genes) were down-regulated in A549 from α11^-/-^/A549 spheroids in comparison with α11^+/+^/A549 spheroids **(Fig. S4**). The top 10 up- or down-regulated genes are listed in **Fig. 5A**. After preliminary analysis by qRT-PCR (data not shown), we decided to focus on the up-regulated gene TIMP2 and down-regulated gene CXCL5. The expression changes at RNA levels for these two genes were further validated by qRT-PCR (**Fig. 5B** **).** The total RNA used for qRT-PCR validation of microarray result was isolated in 3 independent experiments distinct from the total RNA used in microarray analysis.

### Functional validation of CXCL5 as a modulator of proliferation and cell migration

**Figure 6:**
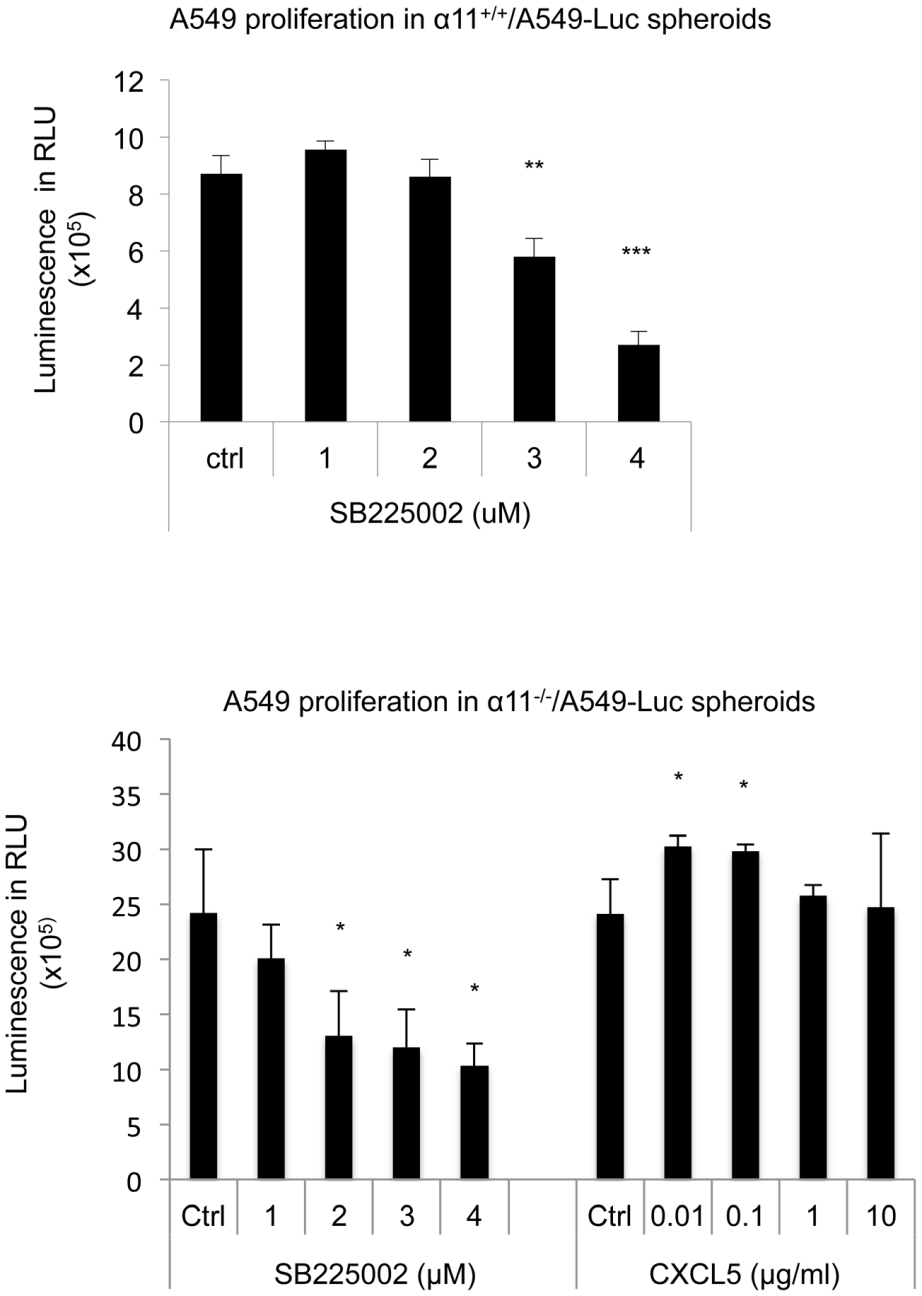
CXCL5 regulates A549 proliferation in heterospheroids. Heterospheroids were prepared with α11^+/+^ or α11^-/-^ MEFs, mixed with A549 cells stably expressing firefly luciferase (A549-Luc). Proliferation of A549 cells was measured by luciferase assay in presence of CXCL5 receptor inhibitor (SB225002) in α11^+/+^/A549 spheroids (upper graph) and in presence of recombinant CXCL5 or SB225002 in α11^-/-^/A549 spheroids (lower graph). Three independent experiments were performed and results from one representative experiments are shown. Error bars represent standard deviation. * p<0.05, ** p<0.01, *** p<0.001.

To determine if CXCL5 in an autocrine manner takes part in regulation of proliferation in the 3D spheroids, we examined the effect of blocking CXCL5 function in α11^+/+^/A549 heterospheroids. CXCL5 binds to the CXCR2 receptor, which is inhibited with the small inhibitor SB225002. At a concentration of 2 µM the inhibitor reduced A549 cell proliferation in the α11^-/-^/A549 heterospheroids, whereas no effect was observed in corresponding α11^+/+^/A549 heterospheroids (**Fig. 6**), suggesting that in the spheroids lacking the fibroblast α11β1 integrin, signaling via the CXCL5/CXCR2 axis is diminished. At 4 µM the inhibitor effectively reduced proliferation in both types of spheroids.

**Figure 7:**
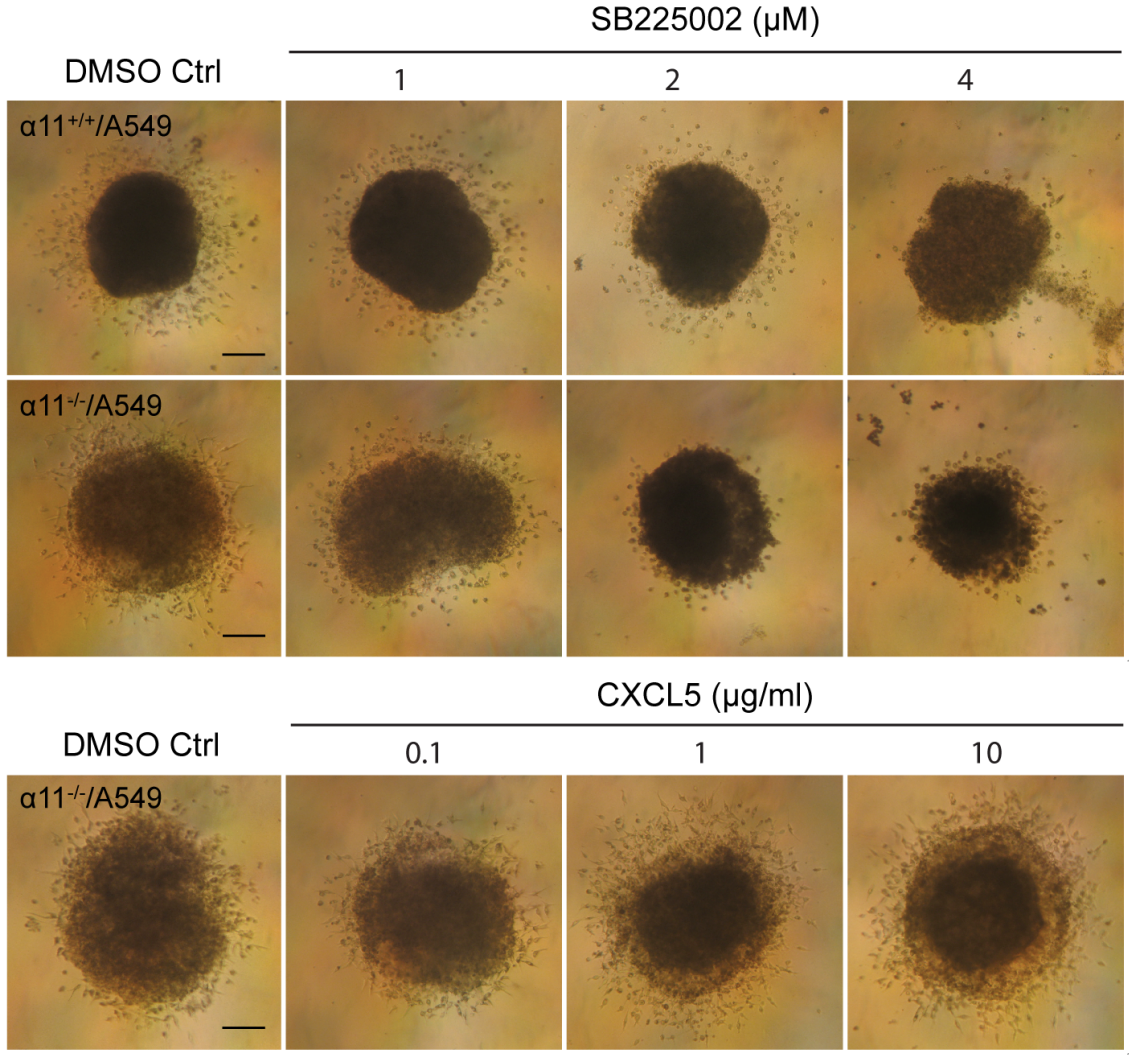
CXCL5 regulates heterospheroid invasion in 3D collagen gels. Six-day-old spheroids were embedded in 3D collagen gels (1 mg/ml collagen type I) in the presence of DMSO (control) or different concentrations of the CXCL5 receptor inhibitor SB225002 (upper graph) or recombinant CXCL5 (lower graph). Six spheroids were imbedded into the collagen gels under each condition. Invasion of the cells from the spheroids into the collagen gels was observed under an inverted phase contrast microscope and photographed after 48 h. Size bar  = 100 µm.

The CXCR2 inhibitor was furthermore added in experiments where cell migration onto a collagen I coated surface was analyzed. The inhibitor (at 2 µM) reduced cell migration in both types of spheroids. Furthermore, addition of recombinant CXCL5 increased cell migration from α11^-/-^/A549 spheroids to the same level as that seen from α11^+/+^/A549 spheroids (**Fig.7**).

## Discussion

Spheroids constitute a model where one can study cell communication in a 3D environment [31], [32], [33]. Most studies have been performed with one cell type in homospheroids, but more and more studies are moving into multicellular spheroids composed of 2 or more cell types, herein referred to as heterospheroids [18], [27], [34], [35], [36]. In studies of tumor cell properties, heterospheroids composed of tumor cells/fibroblasts [17], [35], [37], tumor cells/endothelial cells [31] or preformed heterospheroids where inflammatory cells are added [38], have been used. Heterotypic spheroids composed of tumor and stroma cells have been suggested to represent the early tumor-stroma interactions occurring in the avascular tumor initiation step [39].

Not all cell types form spheroids when put in suspension. A number of studies have been performed to understand the mechanism whereby they form and the molecules involved. These studies have formed the basis for some general models where one basically can distinguish between small and large spheroids [32], [33]. The large spheroids have been characterized as being composed of an inner necrotic core, an intermediate quiescent cell layer and an outer cell layer with proliferating cells [40], [41]. The smaller spheroids lack the necrotic core. In both models it has been postulated that cell migration of proliferating cells toward the oxygen and nourishment-rich spheroid periphery in combination with chemotactic signals are the driving force for the directional migration towards the surface of spheroids. Interestingly, spheroids formed with cells lacking HIF-1α are not much different from spheroids formed from wildtype cells, suggesting a limited role of hypoxia in these models [40]. Other molecules shown to be expressed in a hypoxia- dependent manner, display an expression restricted to the center of large spheroids [42]. Measurements of oxygen levels in spheroids of different sizes [43], suggests that oxygen gradients are relatively small in small spheroids, but considerable in spheroids with a necrotic core. In our experimental set-up we are below the limit where necrotic cores have been observed.

Regarding the detailed molecular mechanism of the intercellular connections that leads to spheroid formation, integrin and cadherin mediated cell adhesion have been shown to regulate spheroid formation (primarily studied in homospheroids). Human dermal fibroblasts and CHO cells form spheroids *in vitro* in an α5β1- and fibronectin- dependent manner and represent examples of the importance of ECM for spheroid integrity [44], [45]. Studies with different tumor cell lines show that carcinoma cells form homospheroids in E-cadherin [46], β1 integrin [44], [45] or both E-cadherin- and β1 integrin-dependent mechanisms, illustrating the importance of both cell-cell and cell-ECM contacts for spheroid formation. Much less is known about the factors influencing spheroid formation in mixed spheroids composed of multiple cell types and the molecules involved in cell communication in the heterospheroid microenvironment.

Heterospheroids can form by mixing preformed homospheroids [12] or by mixing both cell types at different ratios prior to spheroid formation step [27]. A remarkable property of heterospheroids is that cell types sort out, so that tumor cells and fibroblasts cluster independently, thought to reflect the different set-ups of cell surface receptors on the different cell types. Similar as in [27], A549 cells did not spontaneously form spheroids when put in suspension, indicating that the cadherin and integrin linkages are weak under these experimental conditions. However, when A549 were mixed with fibroblasts they did form regular spheroids.

In the current study we demonstrate that α11^-/-^/A549 heterospheroids, lacking α11 integrin on the fibroblasts, exhibit lower interstitial fluid pressure. Previous studies have suggested that tumor stroma stiffness can be mediated by LOX-mediated crosslinking [4]. Our data suggest that another way to modulate the matrix, i.e. the reorganization of the collagen matrix, is determined by the type and levels of the expressed integrins, in turn influencing interstitial fluid pressure and also tissue stiffness. This is in agreement with previous data where we demonstrated that dermal α11β1 has a role in regulating interstitial fluid pressure [7]. Our data implicate α11β1 as an organizer of the matrix in a tumor cell-containing microenvironment and further suggest that α11-expressing fibroblasts may contribute to maintaining the tensional homeostasis in the tumors by contracting the secreted collagen matrix.

In our experimental set-up, microarray analysis identified CXCL5 as one molecule that appears to be differently regulated by the presence of α11 on the fibroblasts. CXCL5 is a chemokine, which via its receptor CXCR2 has been shown to regulate cell proliferation, cell migration and invasion [47], [48]. In the tumor context CXCR2 has been shown to stimulate neutrophil invasion [38], [49] and angiogenesis [50], and to prevent stress induced apoptosis of tumor cells [51]. The role of CXCL5/CXCR2 on tumor cells themselves has been less studied, but a recent study suggests an important role of CXCL5 in lung adenocarcinomas in promoting invasion and metastasis [52]. Another recent study suggests that CAF-derived Il-1β stimulates cholioangiocarcinoma cells to produce CXCL-5, which stimulates their invasion [53]. In yet another study CXCL5 expression was directly dependent on β8 integrin expression and signaling [54]. Interestingly, TGF-β has been shown to down-regulate CXCL5 whereas epithelial loss of TGFB2 results in increased expression of CXCL5 [54], [55], [56]. Our finding that SB431542 was ineffective in blocking α11^+/+^/A549 spheroid invasion agrees with the recent studies suggesting that A549 produce little bioactive TGF-β[26]. Further studies are needed to elucidate the nature of the α11β1-dependent fibroblast signal that increases autocrine CXCL5 expression in A549 cells.

In summary, the fibroblast α11β1 appears to orchestrate cell adhesive events in spheroids, both in fibroblasts and A549 cells. We suggest that in the forming spheroid, the more rapidly proliferating A549 cells move towards cell periphery and that the A549 interaction with α11-positive fibroblasts leads to secretion of CXCL5. CXCL5 by binding to CXCR2 on A549 cells increase proliferation and cell migration. Once the fibroblasts have deposited a collagen matrix, α11β1 mediates contraction of the collagen I matrix, leading to increased interstitial fluid pressure in the spheroids, consolidated cell communications and resulting in a more compact spheroid structure.

We propose that integrin α11β1, expressed by stromal fibroblasts, is an interesting therapeutic candidate molecule with the potential to sense and regulate matrix stiffness and thus interstitial tissue pressure within the tumor. Our data suggests that blocking the mechanosensitive/mechanostransducer α11β1 integrin will increase the effectiveness of chemotherapy.

## Supporting Information

Figure S1 **Integrin α11 expression in mouse embryonic fibroblasts (MEFs).** Western blotting was performed on the MEFs using antibodies against mouse integrin α11 (A) and against human integrin α11 (B) to verify the expression of α11 on α11^+/+^MEFs and on α11^-/-/α11^MEFs, respectively.
(TIF)

Figure S2 **A549 cell segregation in 6-day-old heterospheroids.** Heterospheroids were prepared by liquid overlay method. Six-day-old α11^+/+^/A549 heterospheroids (A, B) and α11^-/-^/A549 heterospheroids (C, D) were double-stained with anti-human cytokeratin 7. Pictures were taken under the fluorescence microscope with 10× (A, C) and 20× (B, D) magnifications.
(TIF)

Figure S3 **TGF-βRI inhibitor SB431542 has no effect on α11^+/+^/A549 heterospheroid invasion in 3D collagen gels.** Six-day-old α11^+/+^/A549 heterospheroids were embedded in 3D collagen gels (3 mg/ml collagen type I) and incubated for up to 72 hours with DMEM with 2% FCS in the presence of DMSO control (upper panel) or TGF-βRI inhibitor SB431542 (lower panel). Invasion of the cells from the heterospheroids into the collagen gels was observed under an inverted phase contrast microscope and photographed at the time points as indicated.
(TIF)

Figure S4 **Cluster two-dimensional expression profile of the 160 genes differentially expressed between 6 α11^+/+^/A549 and 6 α11^-/-^/A549 heterospheroid samples in microarray analysis.** Red: up-regulated genes (136 genes) in α11^-/-^/A549 spheroids versus α11^+/+^/A549 spheroids; Green: down-regulated genes (24 genes) in α11^-/-^/A549 spheroids versus α11^+/+^/A549 spheroids.
(TIF)

Table S1 **List of qRT-PCR primers used for validating the Microarray data.** qRT-PCR validation was performed on 4 selected genes using both human and mouse specific primers. Listed are the sequences of the qRT-PCR primers (13 pairs in total including 5 pairs of primers for the reference genes) and the lengths of the amplicons.
(TIF)
