# Supplementary figures and images for: Fibroblast α11β1 Integrin Regulates Tensional Homeostasis in Fibroblast/A549 Carcinoma Heterospheroids
Source: PLoS One. 2014 Jul 30;9(7):e103173. doi: 10.1371/journal.pone.0103173 (PMC4116160; doi:10.1371/journal.pone.0103173)

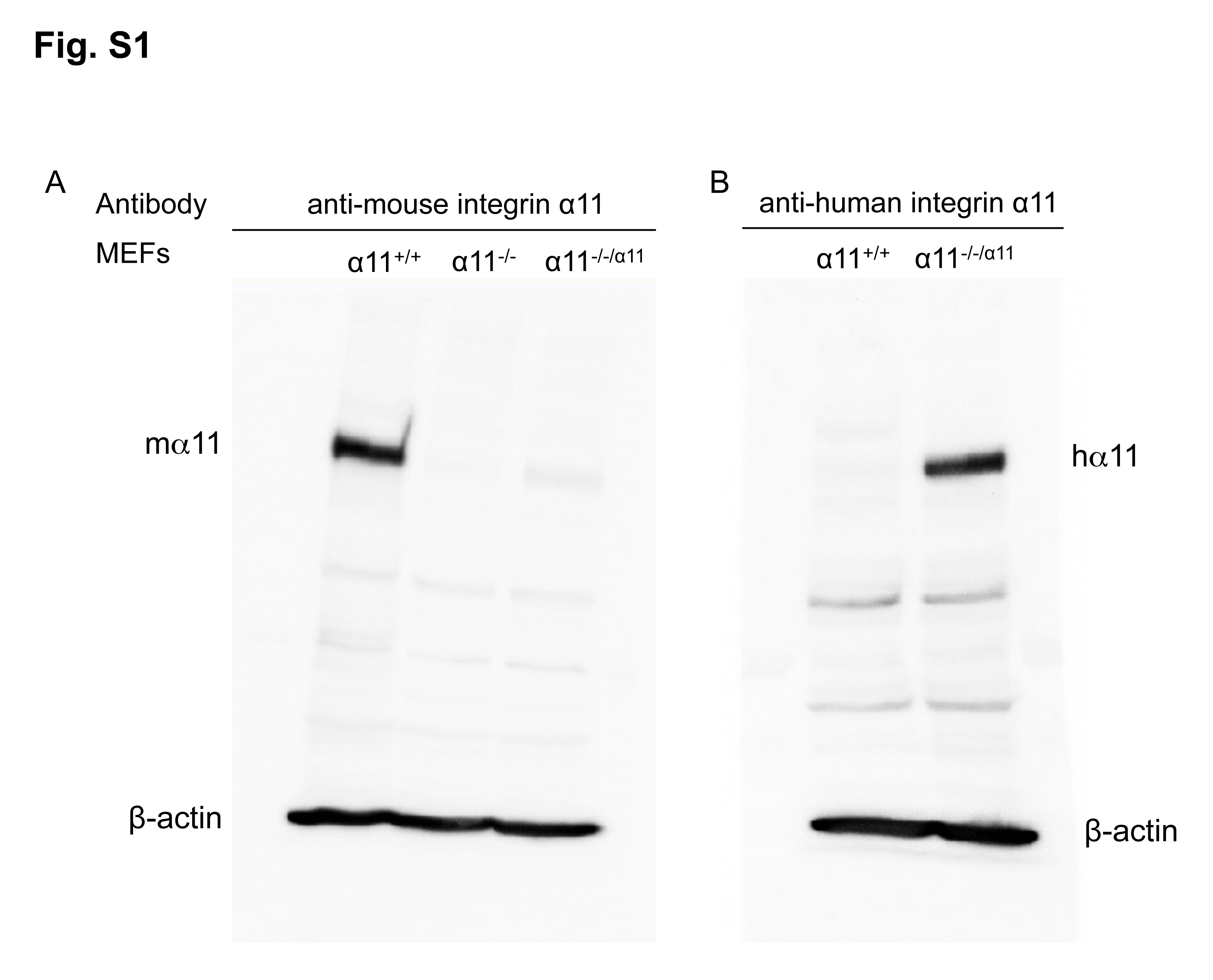

Supplement: Figure S1 — Integrin α11 expression in mouse embryonic fibroblasts (MEFs). Western blotting was performed on the MEFs using antibodies against mouse integrin α11 (A) and against human integrin α11 (B) to verify the expression of α11 on α11+/+MEFs and on α11-/-/α11MEFs, respectively. (TIF) [file pone.0103173.s001.tif]

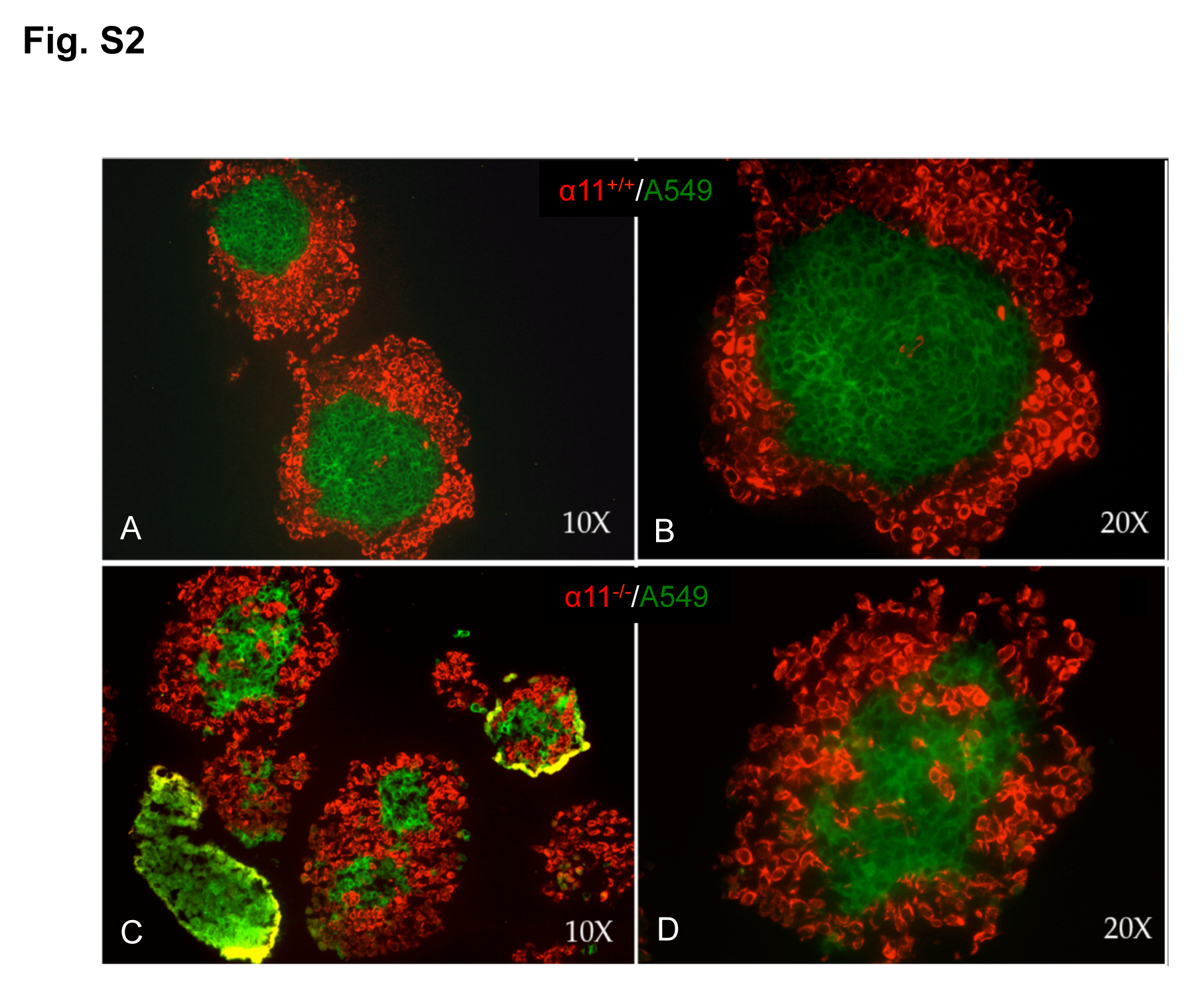

Supplement: Figure S2 — A549 cell segregation in 6-day-old heterospheroids. Heterospheroids were prepared by liquid overlay method. Six-day-old α11+/+/A549 heterospheroids (A, B) and α11-/-/A549 heterospheroids (C, D) were double-stained with anti-human cytokeratin 7. Pictures were taken under the fluorescence microscope with 10× (A, C) and 20× (B, D) magnifications. (TIF) [file pone.0103173.s002.tif]

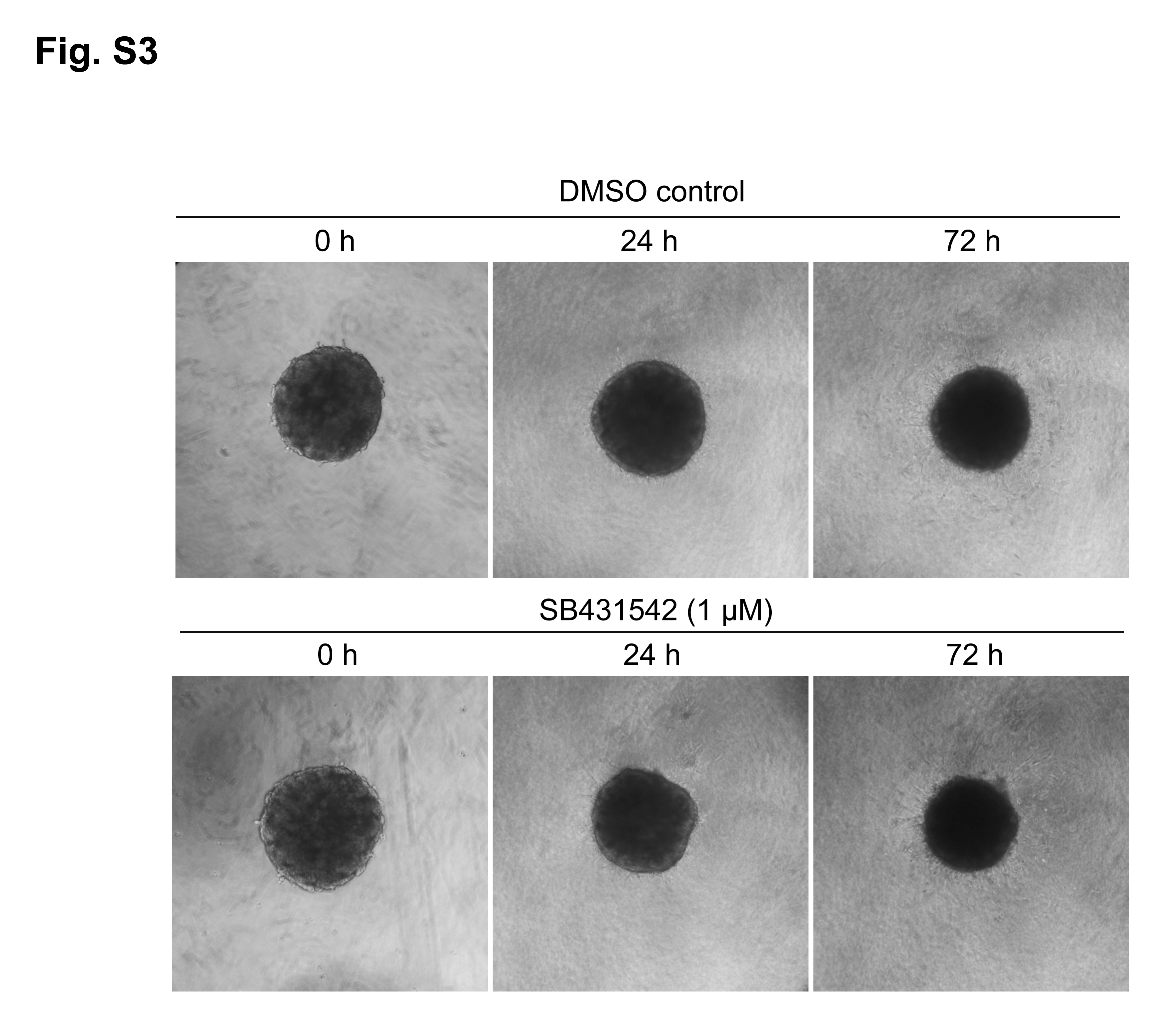

Supplement: Figure S3 — TGF-βRI inhibitor SB431542 has no effect on α11+/+/A549 heterospheroid invasion in 3D collagen gels. Six-day-old α11+/+/A549 heterospheroids were embedded in 3D collagen gels (3 mg/ml collagen type I) and incubated for up to 72 hours with DMEM with 2% FCS in the presence of DMSO control (upper panel) or TGF-βRI inhibitor SB431542 (lower panel). Invasion of the cells from the heterospheroids into the collagen gels was observed under an inverted phase contrast microscope and photographed at the time points as indicated. (TIF) [file pone.0103173.s003.tif]

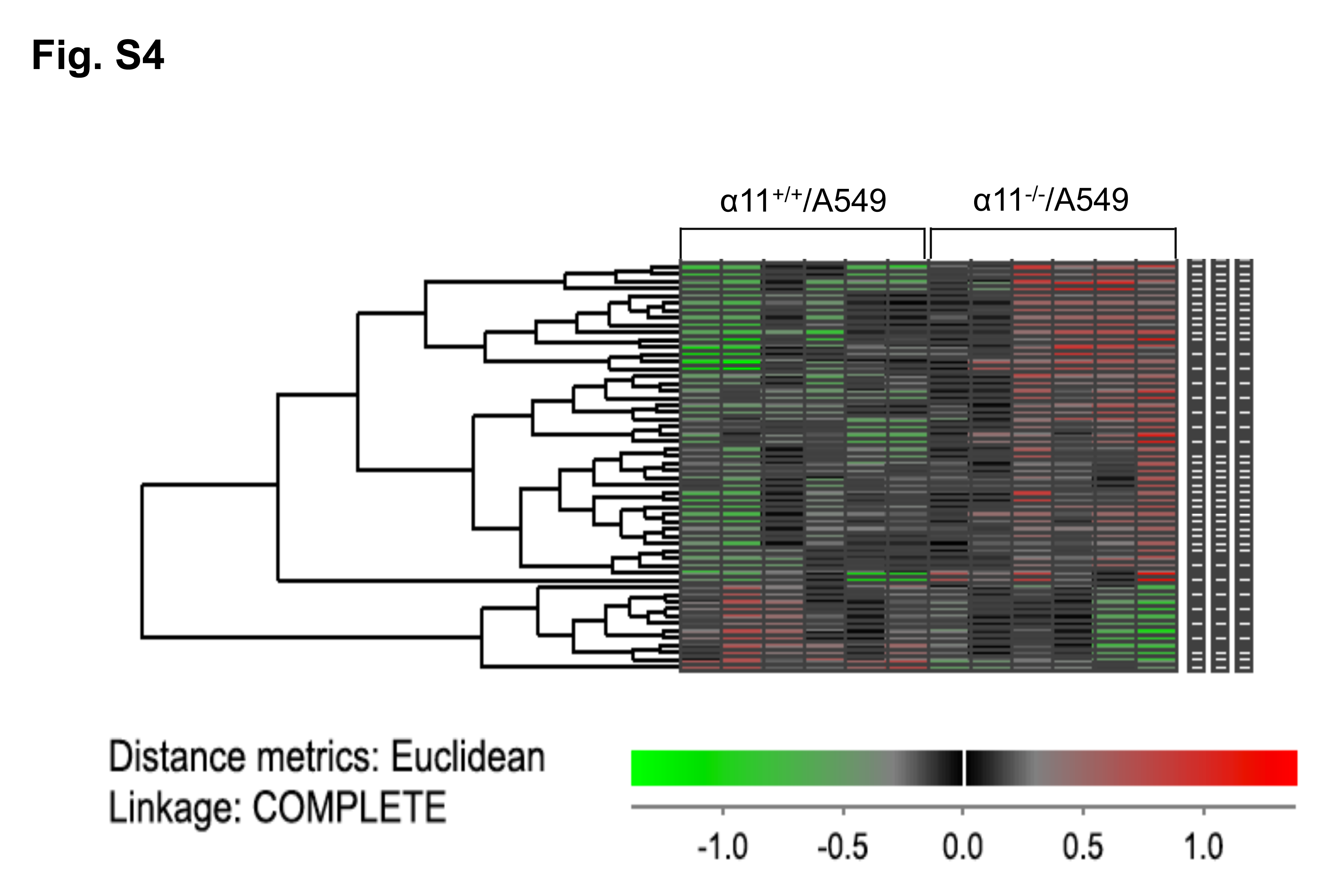

Supplement: Figure S4 — Cluster two-dimensional expression profile of the 160 genes differentially expressed between 6 α11+/+/A549 and 6 α11-/-/A549 heterospheroid samples in microarray analysis. Red: up-regulated genes (136 genes) in α11-/-/A549 spheroids versus α11+/+/A549 spheroids; Green: down-regulated genes (24 genes) in α11-/-/A549 spheroids versus α11+/+/A549 spheroids. (TIF) [file pone.0103173.s004.tif]

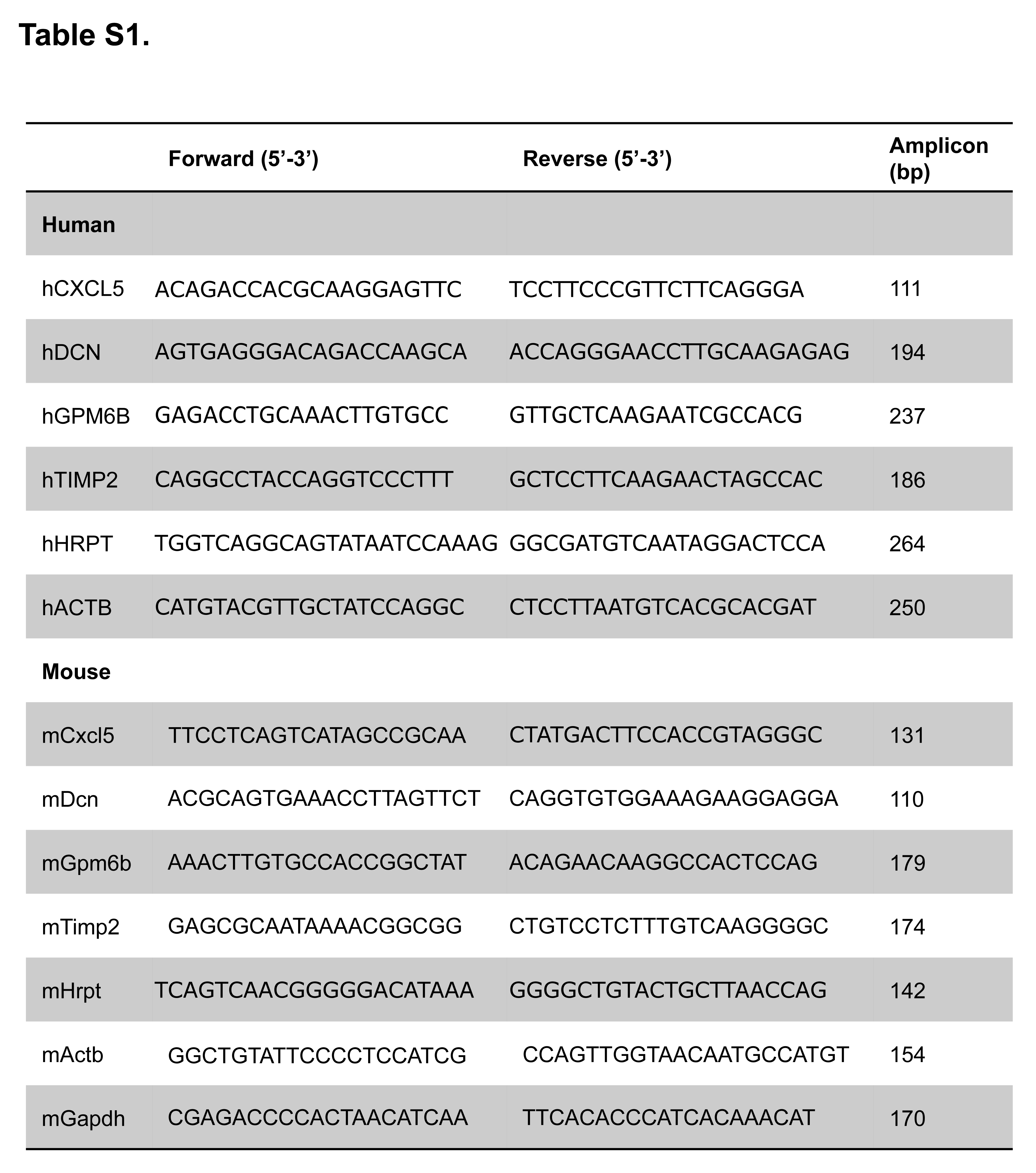

Supplement: Table S1 — List of qRT-PCR primers used for validating the Microarray data. qRT-PCR validation was performed on 4 selected genes using both human and mouse specific primers. Listed are the sequences of the qRT-PCR primers (13 pairs in total including 5 pairs of primers for the reference genes) and the lengths of the amplicons. (TIF) [file pone.0103173.s005.tif]
